# Parasites may exit immunocompromised northern pig-tailed macaques (*Macaca leonina*) infected with SIVmac239

**DOI:** 10.24272/j.issn.2095-8137.2018.015

**Published:** 2018-02-09

**Authors:** Tian-Zhang Song, Ming-Xu Zhang, Yu-Jie Xia, Yu Xiao, Wei Pang, Yong-Tang Zheng

**Affiliations:** 1 Key Laboratory of Animal Models and Human Disease Mechanisms of the Chinese Academy of Sciences/Key Laboratory of Bioactive Peptides of Yunnan Province, Kunming Institute of Zoology, Chinese Academy of Sciences, Kunming Yunnan 650223, China; 2 University of Chinese Academy of Sciences, Beijing 100049, China; 3 Kunming Primate Research Center, Kunming Institute of Zoology, Chinese Academy of Sciences, Kunming Yunnan 650223, China

**Keywords:** AIDS, Immunocompromised, Northern pig-tailed macaque, Parasite, SIVmac239

## Abstract

Parasites can increase infection rates and pathogenicity in immunocompromised human immunodeficiency virus (HIV) patients. However, *in vitro* studies and epidemiological investigations also suggest that parasites might escape immunocompromised hosts during HIV infection. Due to the lack of direct evidence from animal experiments, the effects of parasitic infections on immunocompromised hosts remain unclear. Here, we detected 14 different parasites in six northern pig-tailed macaques (NPMs) before or at the 50th week of simian immunodeficiency virus (SIV) infection by ELISA. The NPMs all carried parasites before viral injection. At the 50th week after viral injection, the individuals with negative results in parasitic detection (i.e., 08247 and 08287) were characterized as the Parasites Exit (PE) group, with the other individuals (i.e., 09203, 09211, 10205, and 10225) characterized as the Parasites Remain (PR) group. Compared with the PR group, the NPMs in the PE group showed higher viral loads, lower CD4^+^ T cells counts, and lower CD4/CD8 rates. Additionally, the PE group had higher immune activation and immune exhaustion of both CD4^+^ and CD8^+^ T cells. Pathological observation showed greater injury to the liver, cecum, colon, spleen, and mesenteric lymph nodes in the PE group. This study showed more seriously compromised immunity in the PE group, strongly indicating that parasites might exit an immunocompromised host.

## INTRODUCTION

Acquired Immune Deficiency Syndrome (AIDS), induced by human immunodeficiency virus (HIV), was first recognized in 1981 and has emerged from initially being a concern among high-risk groups to representing a worldwide pandemic (Jeang et al., 2007). According to estimates by WHO and UNAIDS, 36.7 million people were living with HIV globally at the end of 2015. That same year, approximately 2.1 million people became newly infected and 1.1 million died of HIV-related causes. As HIV specifically acts on the immune system, destroying or impairing its function, the most common causes of death for HIV patients are various pathogenic infections (including fungal, viral, bacterial, and parasitic infections) and cancer (Bonnet et al., 2005; Antiretroviral Therapy Cohort Collaboration, 2010; Palladino et al., 2011). 

Both parasites and HIV infection have major effects on the host immune system, and co-infection is widespread (Bentwich, 2000). Helminths can induce a Th2 bias, leading to suppression of Th1 responses to HIV, as well as to expansion of Th2 lymphocytes that are more susceptible to HIV infection (Bentwich et al., 1999). Several epidemiological and systematic studies claim that deworming is a preventive and possibly therapeutic measure in patients co-infected with helminths and HIV (Borkow & Bentwich, 2006; Walson et al., 2009; Walson & John-Stewart, 2007), thus indicating the potential side effect of parasites. However, a more recent meta-analysis showed that deworming has little or no favorable effect on HIV treatment (Means et al., 2016). Unfortunately, direct experimental evidence from animal models is still missing, and the relationship between parasites and HIV remains complex and unclear.

Non-human primates (NHPs) are crucial animal models for understanding the pathogenesis of HIV infection (Karlsson et al., 1997; Letvin, 1992). Simian immunodeficiency virus (SIV) infections in rhesus macaques and northern pig-tailed macaques (NPMs) result in an immunocompromised and AIDS-like disease similar to that seen in HIV patients, as established in our laboratory (Pang et al., 2017; Zhang et al., 2017a, b). In this study, NPMs with different parasitic loads after infection with SIVmac239 were used to detect immunological and pathological differences to reveal the relationship between viral infection and parasites.

## MATERIALS AND METHODS

### Ethics statement

All animal experiments were performed according to the guidelines of the Committee on Animals of the Kunming Institute of Zoology, Chinese Academy of Sciences (approval number: SYDW-2015023). NPMs included in this study were housed at the Kunming Primate Research Center under the guidance of the American Association for Assessment and Accreditation of Laboratory Animal Care (AAALAC).

### Animals and sample collection

After evaluating each individual for B virus, SIV, STLV, and SRV by PCR, six negative male NPMs (5–7 years old, numbered 08247, 08287, 09203, 09211, 10205, and 10225) were enrolled in this study. Each NPM was inoculated with 2 mL of normal saline containing a 3 000 50% tissue culture infection dose (TCID_50_) of SIVmac239 into the posterior tibial vein. Peripheral blood was collected into an EDTAK_2_ vacutainer by venipuncture. Subsequently, peripheral blood mononuclear cells (PBMCs) were separated by Ficoll density gradient centrifugation (2 500 r/min, 20 min). PBMCs and plasma were stored in liquid nitrogen and frozen at −80 °C, respectively. Ketamine hydrochloride (0.2 mL/kg) was used to anaesthetize animals prior to experiments.

### Parasite detection

Pre-infection and at the 50th week after viral infection, plasma from the NPMs was collected and evaluated for parasitic status. In total, 14 types of parasites were included in this study. *Entamoeba histolytica* was detected by thick smear, as described previously (Warhurst & Williams, 1996). *Trypanosoma brucei*, *Cryptosporidium parvum*, *Plasmodium knowlesi*, *Toxoplasma gondii*, *Giardia lamblia*, *Echinococcus granulosus*, *Clonorchis sinensis, Schistosoma japonicum*, *Paragonimus* spp., filarial worms, and *Trichinella spiralis* were detected by their respective parasitic antigen ELISA kits (MEIMIAN, China) following the manufacturer’s instructions. *Spirometra mansoni* and *Angiostrongylus cantonensis* were detected by their respective human antibodies ELISA kits (MEIMIAN, China; JIANLUN, China) following the manufacturer’s instructions. For all ELISA experiments, the Calculate Critical (CUT OFF) value was equal to the average of the negative control well plus 0.15. The well with an OD greater than or equal to the CUT OFF was regarded as a positive result.

### Full blood differential counts

Before separation of PBMCs, 80 µL of blood was removed from the EDTAK_2_ vacutainer. Whole blood differential counts were performed by an auto hematology analyzer (RR-OB101338, MINDRAY, Shenzhen, China) according to the manufacturer’s instructions.

### Plasma viral loads

Viral loads in plasma were measured by quantitative PCR for SIV gag RNA, as described previously (Tian et al., 2015) (SIV F: 5'-TCGGTCTTAGCTCCATTAGTGCC-3'; SIV R: 5'-GCTTCCTCAGTGTGTTTCACTTTC-3'; SIV probe: 5'-CTTCTGCGTGAATGCACCAGATGACGC-3').

### Flow cytometry

Multicolor flow cytometric analysis was performed as described previously (Xia et al., 2009; Zheng et al., 2014). Anti-CD3 PE (clone SP34-2), anti-CD8 PECy7 (clone RPA-T8), anti-CD20 FITC (clone 2H7), anti-CD14 APC (clone M5E2), anti-CD3 APC-Cy7 (clone SP34-2), and anti-HLA-DR APC (clone G46-6) were purchased from BD Pharmingen (Franklin Lakes, New Jersey, USA). Anti-CD4 PerCP (clone OKT4) was purchased from Biolegend (San Diego, CA, USA). Anti-CD38 FITC (clone AT-1) was purchased from STEMCELL (Vancouver, Canada). Anti-PD-1 PE (clone eBioJ105) was purchased from eBioscience (San Diego, CA, USA). Samples were processed on a BD FACSVerse flow cytometer (Franklin Lakes, New Jersey, USA), and data were analysed using FlowJo software (vX.0.7, Tree Star).

### Tissue collection and histopathology

Both 08247 and 09211 died naturally on the 70th and 68th weeks after viral infection, respectively. Tissues were fixed in 10% formaldehyde and later embedded in paraffin. The paraffin-embedded samples were cut into 4-μm-thick sections and stained with hematoxylin-eosin (H&E). All samples were photographed and examined using a LEICA DMI 4000B microscope (Germany).

### Statistical analysis

For all data, GraphPad 6.0 was used to perform statistical analyses. Two-way analysis of variance (ANOVA) was used to compare all data. *P*<0.05 were regarded as statistically significant. 

## RESULTS

### Parasitic infection status and categories

The parasitic infection statuses of the NPMs are shown in Table 1. None of the NPMs was infected with *Plasmodium knowlesi*, *Echinococcus granulosus*, *Clonorchis sinensis*, *Paragonimus* spp., *Angiostrongylus cantonensis*, *Trichinella spiralis*, or *Spirometra mansoni*. Both 08247 and 08287 showed positive parasitic reactions before viral infection, and negative reactions after infection. However, 09203, 09211, 10205, and 10225 exhibited substantially different results. At the 50th week after infection, the parasites detected before viral infection remained, and no new parasites were detected after viral infection. Therefore, we grouped 08247 and 08287 into the Parasites Exit (PE) group and the other four NPMs into the Parasites Remain (PR) group to evaluate the relationship between SIV and parasitic infection.

**Table 1:** Parasitic infection status

No.	SIV Infection (N/Y)	Protozoa					
		*Trypanosoma brucei*	*Cryptosporidium* parvum	*Plasmodium knowlesi*	*Entamoeba histolytica*	*Toxoplasma gondii*	*Giardia lamblia*
**08247**	N	**+**	**+**	**-**	**-**	**+**	**+**
	Y	**-**	**-**	**-**	**-**	**-**	**-**
**08287**	N	**+**	**+**	**-**	**-**	**+**	**+**
	Y	**-**	**-**	**-**	**-**	**-**	**-**
**09203**	N	**+**	**+**	**-**	**-**	**-**	**-**
	Y	**+**	**+**	**-**	**-**	**+**	**-**
**09211**	N	**+**	**-**	**-**	**-**	**-**	**-**
	Y	**+**	**-**	**-**	**-**	**-**	**-**
**10205**	N	**+**	**+**	**-**	**-**	**-**	**-**
	Y	**+**	**+**	**-**	**-**	**-**	**-**
**10225**	N	**+**	**+**	**-**	**+**	**+**	**-**
	Y	**+**	**+**	**-**	**+**	**+**	**+**

### Higher viral loads in the PE group

**Figure 1:**
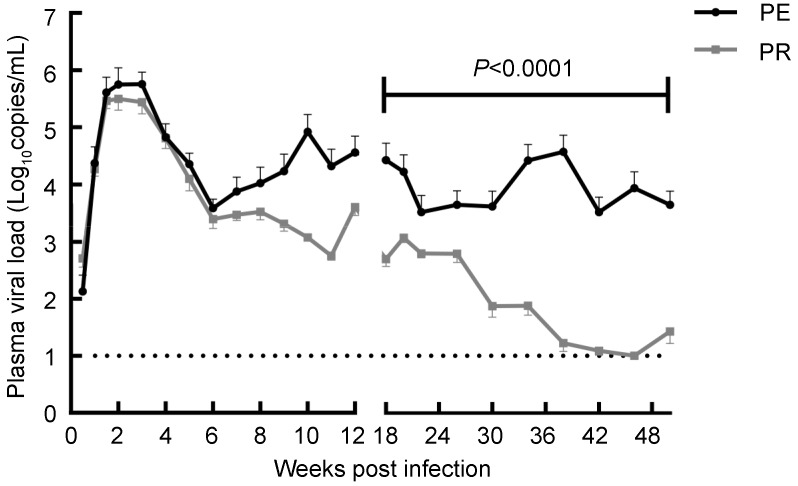
Plasma viral loads in the PE and PR groups

NPMs in both groups had peak plasma viral loads at the second to third week post-infection, after which the viral loads showed a downward trend until the sixth week after injection (Figure 1). Afterwards, the PE and PR groups showed substantially different trends. Viral loads in the PE group stopped decreasing and generally stabilized at 10^4^–10^5^ copies/mL. However, the PR group showed a continuing downward trend, and approached detection limits at the 40th–50th weeks after infection. In this study, the 50 experimental weeks were divided into SIV acute phase and SIV chronic phase at the 12th week post infection to perform additional analysis (Pantaleo et al., 1993b). Compared with the PR group, the PE group showed significantly higher plasma viral loads in the chronic stage (*P*<0.000 1).

### Lower CD4^+^ T cell levels in the PE group

**Figure 2:**
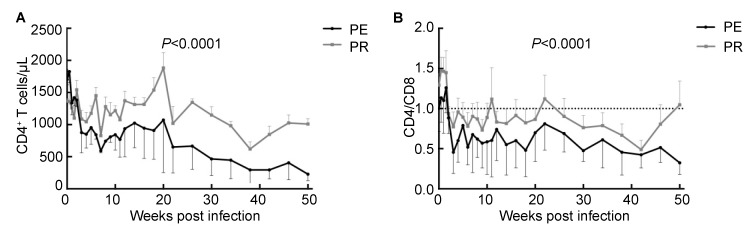
Dynamics of CD4^+^ T cells in peripheral blood

Absolute counts of CD4^+^ T cells, the primary cells infected by HIV or SIV, were evaluated by flow cytometry. Compared with the PR group, NPMs in the PE group had significantly fewer CD4^+^ T cells in peripheral blood (*P<*0.000 1, Figure 2A). This was in accordance with the results in Figure 2B, which show a significantly lower CD4^+^/CD8^+^ T cell rate in the PE group (*P<*0.001). These two results strongly indicated that the NPMs in the PE group had more seriously damaged CD4^+^ T cells.

A: Absolute counts of CD4^+^ T cells; B: Ratio between CD4^+^ T cells and CD8^+^ T cells.

### Immune activation and exhaustion

**Figure 3:**
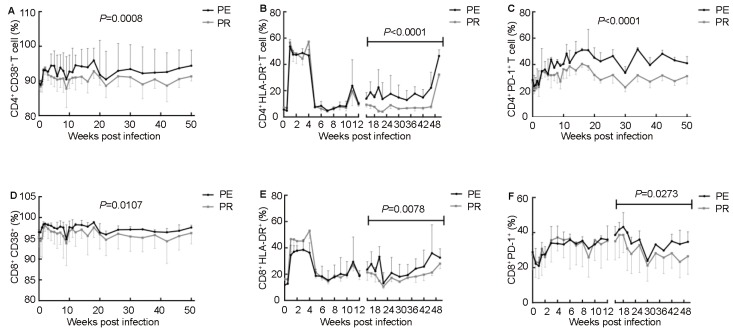
Expression of immune activation and exhaustion markers

Chronic immune activation is proposed to be a key determinant of AIDS pathogenesis (Rajasuriar et al., 2013), and was marked by CD38 and HLA-DR in this study. There were significant differences between the PE and PR groups in CD38 expression during the whole study period and in HLA-DR expression during the chronic phase in CD4^+^ and CD8^+^ T cells (Figure 3A, B, D, E). These data strongly indicated that NPMs in the PE group showed more obvious immune activation than those in the PR group during SIV infection. Additionally, the PE group had a higher level of PD-1 expression in CD4^+^ T cells during the whole study period (*P<*0.000 1) and in CD8^+^ T cells during the chronic phase (*P*=0.027 3), as shown in Figure 3C and 3F respectively. Collectively, these results revealed that NPMs in the PE group showed higher immune activation and more serious exhaustion of CD4^+^ and CD8^+^ T cells.

A, D: Expression of CD38 in CD4^+^ and CD8^+^ T cells; B, E: Expression of HLA-DR in CD4^+^ and CD8^+^ T cells; C, F: Expression of PD-1 in CD4^+^ and CD8^+^ T cells.

### Cell distribution in peripheral blood

**Figure 4:**
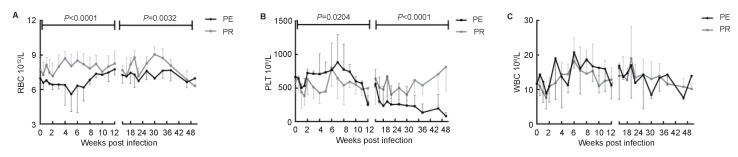
Cell distribution in peripheral blood

The fluctuant cell distribution in peripheral blood was shown in Figure 4. The number of white blood cells showed no significant differences between the two groups (Figure 4C). However, in the SIV acute and chronic phases, NPMs in the PE group showed fewer red blood cells compared with the PR group, as shown in Figure 4A. The number of platelets also significantly declined in the PE group compared with the PR group during the SIV chronic phase (Figure 4B), which is important given that platelet count is a main marker for immunocompromised thrombocytopenic purpura induced by HIV or SIV (O’Bryan et al., 2015).

A: Red blood cell (RBC) counts; B: Platelet (PLT) counts; C: White blood cell (WBC) counts.

### Dynamics of monocytes, B cells, and T cells in peripheral blood

**Figure 5:**
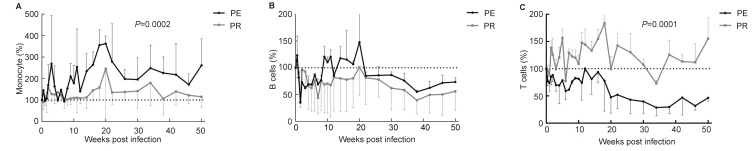
Dynamics of monocytes, B cells, and T cells in peripheral blood

Cell counts before viral infection were calculated as 100%. The dynamics of the three cell types were evaluated, as shown in Figure 5. Monocytes, which play an important role in innate immunity, showed a significant increase in the PE group compared with the PR group (Figure 5A). Conversely, T cells and B cells, markers of acquired immunity, were significantly reduced in the PE group compared with the PR group, as shown in Figure 5B, C.

### Pathological observation of injured organs

**Figure 6:**
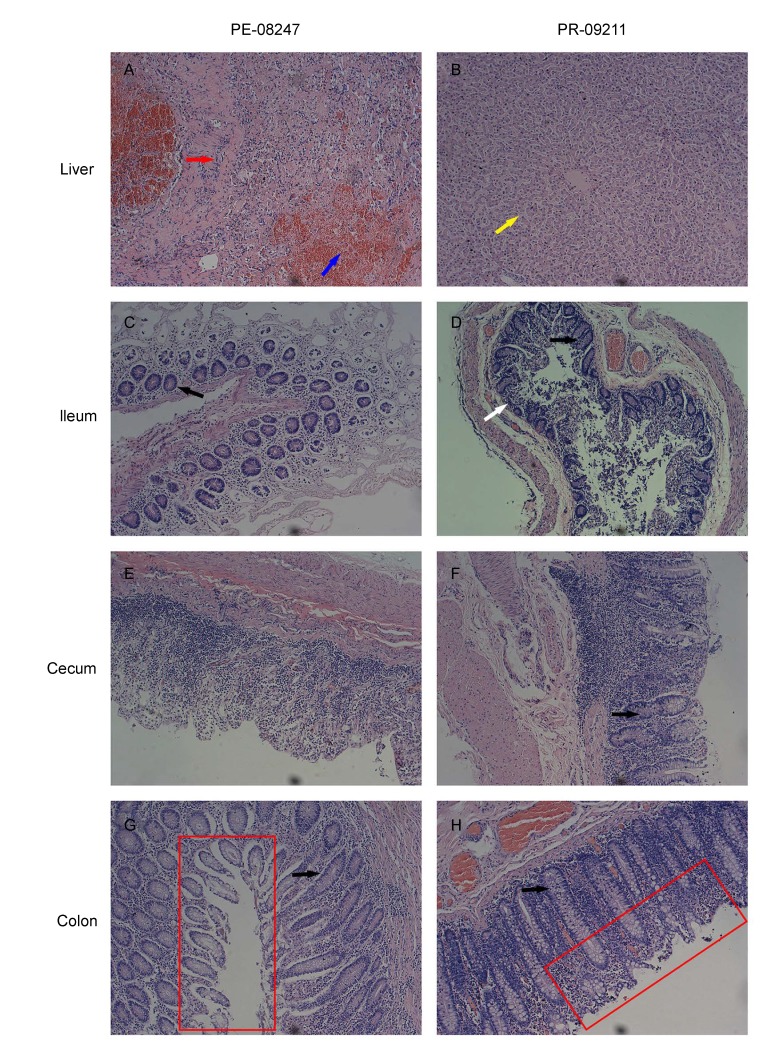
Histological changes in liver, ileum, cecum, and colon

In total, 22 organs from each NPM were collected and stained by H&E, four of which displayed obvious damage, as shown in Figure 6. Compared to PR-09211, which maintained almost normal hepatic lobule appearance, liver damage in PE-08247 was obvious (Figure 6A). Alimentary canal damage is strongly related to chronic immune activation (Griffin, 1990; Mestecky et al., 2009), and was detected in our study. In Figure 6C, D, obvious damage in the ileum of both 08247 and 09211 could be seen. Compared to 09211, PE-08247 demonstrated more serious injuries in the cecum without any observed enteraden and in the colon with a discrete and defective mucosal surface.

A, B: Liver (×200); C, D: Ileum (×100); E, F: Cecum (×100); G, H: Colon (×100). Red arrow: Fibrosis; Blue arrow: Hemorrhage; Yellow arrow: Hepatic lobule; Black arrow: Enteraden; White arrow: Connective tissue exposure; Red rectangle: Mucosal surface.

### Pathological observation of immune organs

**Figure 7:**
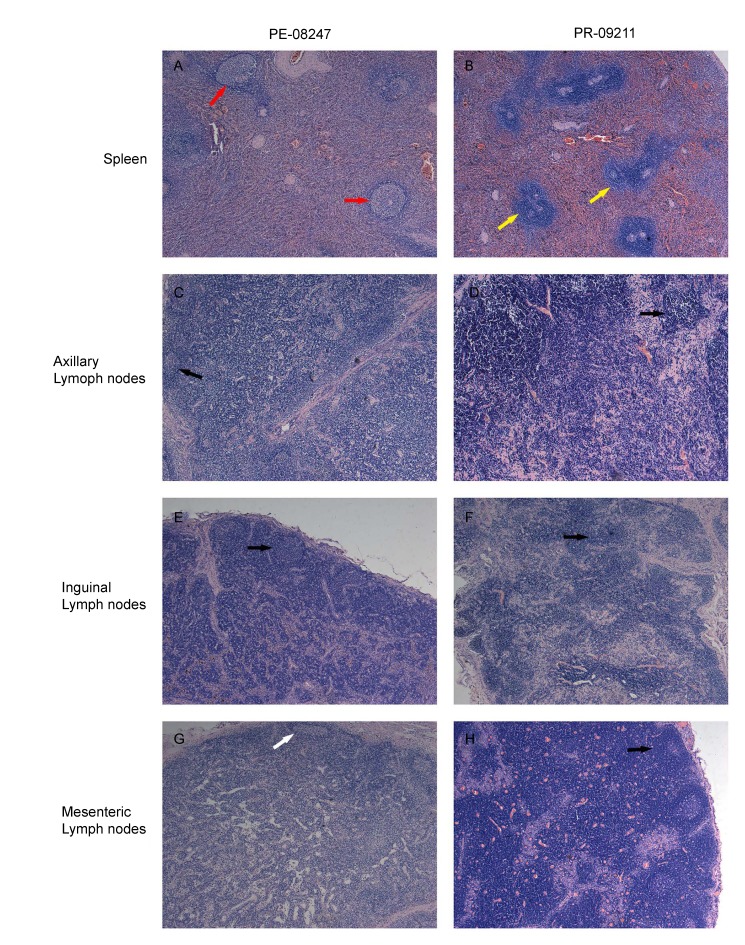
Histological changes in immune organs (×100)

The spleen has no direct connection to the lymphatic system; instead, it collects antigens from the blood and is involved in immune responses to blood-borne pathogens (Murphy & Weaver, 2016). Compared to 09211, the white pulp of 08247 in the PE group had severe fibrosis and decreased lymphocytes, as shown in Figure 7A. Lymph nodes are the most important organ in HIV or SIV infection, spread, and damage to lymphocytes (Pantaleo et al., 1993a). As shown in Figure 7C–F, lymphoid follicles could be observed in all images, and there were no significant differences between the PE and PR groups in axillary and inguinal lymph nodes. However, compared to 09211 in the PR group, mesenteric lymph nodes in 08247 in the PE group were observed with lymphoid follicle fibrosis and decreased lymphocytes in the paracortex, strongly indicating more serious damage in the PE group (Figure 7G).

A, B: Spleen; C, D: Axillary lymph nodes; E, F: Inguinal lymph nodes; G, H: Mesenteric lymph nodes. Red arrow: Abnormal white pulp; Yellow arrow: White pulp; Black arrow: Lymphoid follicle; White arrow: Abnormal lymphoid follicle.

## DISCUSSION

In this study, no parasites were detected in 08247 and 08287 in the PE group at the 50th week after viral infection, although both were positive for parasites before viral injection. In addition, in the PE group, more significant damage to the mucosal barrier, mesenteric lymph nodes, and immune cells were observed.

CD4^+^ T cell counts and viral loads are the most important indices used to predict disease progress and immune system damage (Rowley, 2014). The persistent decrease in CD4^+^ T cell counts with viral load increase represents serious immunological compromise and poor prognosis, which was observed in the PE group. In addition, immune activation is a sensitive index for predicting disease progression and is central to the understanding of HIV-1 disease pathogenesis and progression in association with inflammation (Ipp et al., 2014). Therefore, CD38 and HLA-DR, two markers of immune activation, were detected by flow cytometry in this study, which indicated more significant immune activation in the PE group compared with the PR group.

The loss of enteric mucosal barrier integrity is associated with rapid depletion of enteric lymphocytes, immune activation, and microbial translocation (Brenchley et al., 2006; Douek, 2007). As shown in Figure 6, PE-08247 exhibited serious damage to the mucosal barrier. Unsurprisingly, antigen origin from the translocation of microbes and other bacterial products stimulates mesenteric lymph nodes, resulting in lymphoid tissue hyperplasia and ultimately in the diffuse effacement of lymph node architecture (Lederman & Margolis, 2008), which was observed in the PE group (Figure 7). The decrease in platelets, which is correlated with immunological compromise and virus-related mechanisms, was also detected in the PE group. The significant increase in monocytes and decrease in T cells in peripheral blood indicated changes to the immune system in the PE group. These results showed that NPMs in the PE group were more seriously immunocompromised.

Considering the parasitic detection results in the PE group before and after viral infection, parasites may exit hosts that are seriously immunocompromised. Doenhoff et al. (1986) found that granuloma formation and consequent schistosome egg excretion might be reduced in animal models of immunosuppression. This was supported by subsequent studies in humans (Karanja et al., 1997; Mwanakasale et al., 2003; N’Zoukoudi-N’Doundou et al., 1995). Conversely, another hypothesis suggests that schistosomal infection could be regarded as an immune reconstitution marker following antiretroviral therapy, whereby pathological responses to schistosomes and schistosome eggs are inhibited in advanced HIV disease and deteriorate with immune recovery (De Silva et al., 2006; Fernando & Miller, 2002). Additionally, in contrast to the theory that deworming is a preventive and possible therapeutic measure for patients with co-infection of helminths and HIV (Borkow & Bentwich, 2006; Walson et al., 2009; Walson & John-Stewart, 2007), recent meta-analysis provides stronger evidence that deworming has little or no favorable effect on HIV treatment (Means et al., 2016). These studies suggest that changes in the host induced by HIV or SIV produce adverse effects on parasites, and parasites might not contribute to the progression of HIV disease, matching the hypothesis of Brown et al. (2006).

There were three notable limitations to this study. First, there were only six NPMs enrolled, which might not fully represent the differences between PE and PR groups. Second, feces detection is regarded as the gold standard for the detection of some parasites, particularly intestinal parasites. Unfortunately, we did not collect fecal samples from the six NPMs before viral infection. Therefore, all experiments were performed using antigen or antibody ELISA kits. Finally, tissues were collected from only two NPMs. The other survivors were kept for long-term parasitic experiments. Euthanasia was not performed in this study. However, most of the results in this study showed an identical trend suggesting the conclusions were effective and meaningful.

## CONCLUSIONS

To the best of our knowledge, this study is the first to provide direct evidence on the changes in parasitic infection status before and after SIV injection in an animal model. Both HIV and SIV destroy the host immune system, leading to serious immunological compromise, which enhances the possibility for pathogenic microorganisms, including bacteria, fungi, and viruses, to invade and cause damage. In contrast, our results indicated that parasites may exit an immunocompromised host. These prospective results should be evaluated further with a larger sample size.
